# “You’re dealing with an emotionally charged individual…”: an industry perspective on the challenges posed by medical tourists’ informal caregiver-companions

**DOI:** 10.1186/1744-8603-9-31

**Published:** 2013-07-26

**Authors:** Victoria Casey, Valorie A Crooks, Jeremy Snyder, Leigh Turner

**Affiliations:** 1 Department of Geography, Simon Fraser University, 8888 University Drive, Burnaby, Canada; 2 Department of Health Sciences, Simon Fraser University, 8888 University Drive, Burnaby, Canada; 3 Center for Bioethics, School of Public Health, and College of Pharmacy, University of Minnesota, Minneapolis, USA

**Keywords:** Medical tourism, Informal caregiving, International patient coordinators, Patient, International health care

## Abstract

**Background:**

Patients engage in medical tourism when they privately obtain a medical care abroad. Previous research shows that many medical tourists travel abroad with friends and family members who provide support and assistance. Meanwhile, very little is known about this important stakeholder group, referred to here as caregiver-companions. In this article we examine the challenges that can be posed by caregiver-companions and the overall practice of informal caregiving in medical tourism from an industry perspective. Specifically, we report on the findings of interviews conducted with international patient coordinators (IPCs) who work at destination facilities. IPCs come into regular contact with caregiver-companions in their professional positions and thus are ideally suited to comment on trends they have observed among this stakeholder group as well as the challenges they can pose to medical tourists, health workers, and facilities.

**Methods:**

We conducted 20 semi-structured interviews with 21 IPCs from 16 different facilities across nine countries. Topics probed in the interviews included caregiver-companion roles, IPCs’ and others’ interaction with caregiver-companions, and potential health and safety risks posed to medical tourists and caregiver-companions. Thematic analysis of the verbatim transcripts was employed.

**Results:**

Although most participants encouraged medical tourists to travel with a caregiver-companion, many challenges associated with caregiver-companions were identified. Three themes best characterize the challenges that emerged: (1) caregiver-companions require time, attention and resources; (2) caregiver-companions can disrupt the provision of quality care; and (3) caregiver-companions can be exposed to risks. IPCs pointed out that caregiver-companions may, for example, have a negative impact on the patient through cost of accompaniment or inadequate care provision. Caregiver-companions may also create unanticipated or extra work for IPCs, as additional clients and by ignoring established organizational rules, routines, and expectations. Furthermore, caregiver-companions may be susceptible to stresses and health and safety risks, which would further deteriorate their own abilities to offer the patient quality care.

**Conclusions:**

Although caregiver-companions can pose challenges to medical tourists, health workers, and medical tourism facilities, they can also assist in enhancing best care and offering meaningful support to medical tourists. If caregiver-companions are open to collaboration with IPCs, and particularly in the form of information sharing, then their experience abroad can be safer and less stressful for themselves and, by extension, for the accompanied patients and facility staff.

## Background

Medical tourism involves patients travelling abroad to obtain non-emergency private medical care outside of established cross-boarder care arrangements [1,2]. Because this medical care is private, usually the patient covers the costs of treatment and travel out of pocket [1,3]. Individuals engage in medical tourism for a variety of reasons, including the desire to: obtain procedures not available in their home country, save money on procedures that are not covered by their government or private health insurance, and avoid long wait times for procedures in their home countries [4-7]. Medical tourists from varied socio-economic backgrounds travel from a myriad of countries to obtain procedures in destination countries that are as diverse [1,8,9]. Another noteworthy feature of this globalizing health care industry is that friends and family members often accompany medical tourists abroad [1,9-12]. They may adopt a supportive role during the planning stages, while abroad, and upon return home, and are referred to here as caregiver-companions. In this article we shed light on caregiver-companions’ central role as actors and stakeholders in medical tourism.

Little is known about caregiver-companions in medical tourism and the roles they take on in this global health services practice. In our previous research, it was found that they are an important part of many Canadian medical tourists’ experiences at home and abroad [13]. Despite this, no dedicated research attention has been given to this group. The only published works that offer any depth of insight into the experiences of caregiver-companions are two biographical novels about medical tourists’ journeys written by their caregiver-companions, though in both novels the authors focus heavily on the medical tourists’ experiences and only somewhat on their own [14-16]. Some industry reports e.g. [17,18] and academic studies (e.g. [11-13]) have also attempted to quantify the number of friends and family members who accompany medical tourists abroad, though these pieces do not offer an experiential perspective on caregiver-companions. Otherwise, we have little understanding of the roles played by caregiver-companions, the benefits and challenges they introduce as stakeholders in medical tourism, and the risks they may incur while abroad, among many other aspects of their experiences.

It is important to have a better understanding of caregiver-companions because they embody the informal caregiving (i.e., unpaid care by untrained individuals) component in the medical tourism industry. Research consistently shows that informal caregivers - usually patients’ friends and family – play a key role in maintaining patients’ health and wellness [19-21]. While a competent caregiver may improve a patient’s health, one who is less prepared and supported may be detrimental to it [22,23]. Providing informal care can also have a negative impact on the health of the caregiver. For example, informal caregivers are susceptible to caregiver burden due to stress and burnout, which can present as anxiety, depression, and other mental health issues [24-30]. Having access to meaningful supports such as information or respite care providers, however, can assist with preventing the onset of such burden, and lead to a more positive caregiving experience [31,32]. In this paper, we provide a glimpse into the unique practice of *transnational* informal caregiving in the context of medical tourism.

The existing literature has served to show that there are many actors or stakeholder groups that, together, enable the practice of medical tourism. Patients receive care while surgeons and other physicians provide it, facilitators aid with connecting patients to hospitals and making travel bookings, and policy-makers and government officials work to ensure that destination countries have adequate infrastructure to receive these bookings [33,34]. International patient coordinators (IPCs) constitute yet another such group. They are professionals who work at or with medical tourism destination facilities to coordinate the on-site care of medical tourists. Among many other activities, they often create itineraries for patients and serve as a point of contact for questions or concerns. Their responsibilities typically begin before a patient’s booking is secured and continue until after a patient has returned home. Here we examine informal caregiving in medical tourism from the perspective of IPCs. This allows us to obtain a breadth of insight, which we believe is important in order to identify issues that warrant subsequent investigation in depth. Out of any other medical tourism stakeholder group apart from the medical tourists themselves, IPCs have the most interaction with each caregiver-companion. In fact, it is usual for IPCs working in larger facilities to meet hundreds of caregiver-companions every year, which heightens their knowledge of trends among this medical tourism stakeholder group. It is for this reason that in the current article we examine facets of the caregiver-companion experience from the perspectives of IPCs. By interviewing IPCs from a range of medical tourism destination facilities across several countries, herein we are able to offer insights that have emerged from IPCs’ interactions with many hundreds if not thousands of caregiver-companions and medical tourists alike.

In this article we present the findings of an analysis that examines the challenges that caregiver-companions can pose to IPCs, destination medical tourism hospitals/clinics and their staff, medical tourists, and even themselves from the perspectives of IPCs. It is important to note that the majority of IPCs we spoke with encouraged medical tourists to bring a friend or family member abroad with them because they commonly provide help and companionship that, they observed, positively affects medical tourist well-being. However, they also identified challenges that can be introduced with the presence of caregiver-companions. We believe that articulating these challenges helps to shed light on issues posed by the practice of informal caregiving in the global health services practice of medical tourism and the presence of caregiver-companions in destination facilities. To accomplish this, in the section that follows we outline the study design. Following this, we present the findings of a thematic analysis that identifies three characterizations of the challenges that caregiver-companions pose, in that they can: (1) require time, attention, and resources; (2) disrupt provisions of quality care; and (3) be exposed to risks. We then move to offer a discussion that contrasts the findings of the thematic analysis against existing findings in the medical tourism and informal caregiving literatures while also offering comments on directions for future research.

## Methods

This analysis is one component of a larger multi-method study. The purpose of the multi-method study is to gain an understanding of the experiences of Canadian medical tourists’ caregiver-companions through gathering and analyzing their first-hand accounts and those of other stakeholders. Stakeholder groups consulted in the study are: Canadian medical tourists, Canadian medical tourists’ caregiver-companions, Canadian medical tourism facilitators, and IPCs in various countries. In this paper, we focus exclusively on the IPC dataset. IPCs are an incredibly valuable informant group for the study because they interact routinely and closely with caregiver-companions, as well as medical tourists, and so are well placed to provide a breadth of insight into caregiver-companions’ experiences. It is important to note that although the larger study to which this analysis contributes has a focus on outbound medical tourism by Canadians, the IPCs we interviewed were not asked to comment specifically on Canadian patients but, instead, spoke on broad trends about all the caregiver-companions with whom they have interacted.

### Recruitment

Recruitment commenced upon receiving approval for the study from the Research Ethics Board at Simon Fraser University. Following receipt of this approval, we sought to recruit IPCs from a diverse range of countries and facilities to participate in phone and Skype interviews. Our target sample size was twenty, which is the number of IPCs we anticipated needing to speak with in order to achieve purposeful diversity in the sample. We used several methods to recruit participants, namely: (1) emailing letters of invitation to hospitals whose websites mentioned IPCs, IPCs found in online medical tourism directories, and IPCs who had posted on online forums; (2) snowballing out from existing participants; and (3) disseminating calls for participants through our team’s networks and online forums and magazines. All emails and advertisements included the interviewer’s contact information and indicated that interviews could be conducted in English or French (a request for a Spanish-language interview was also accommodated).

Upon receiving responses from potential participants, they were emailed a study information sheet and eligibility was confirmed. Eligibility required that participants were indeed IPCs and were based in or worked with medical tourism clinics or hospitals that offered surgical procedures that did not involve third parties (e.g., organ transplantation). Not all potential participants used the job title IPC, and so they had to indicate that in their jobs they were present in the facility with the medical tourist, made care and other arrangements, and assisted clients in a non-clinical capacity. In order to enable as much diversity among the sample to be captured, no more than 3 people from a single facility were interviewed. For those who met these eligibility requirements, an interview was scheduled at a time of their convenience.

### Data collection

Telephone and Skype interviews were conducted from July to October, 2012. Interviews were semi-structured in order to ensure that core issues were consistently probed and to allow participants to introduce topics that captured unanticipated experiences and insights. To ensure consistency, the first author conducted all the interviews apart from one. That interview was with a Spanish-speaking participant and was conducted by a knowledgeable collaborator.

All phone interviews started by obtaining verbal consent, in which confidentiality to the furthest extent of the law was assured. Verbal consent was chosen over written consent to minimize the logistical difficulties inherent in the international scope of our study. Interviews generally lasted from 45 to 75 minutes. They covered a range of topics: (1) caregiver-companion characteristics, (2) interactions between caregiver-companions and hospital staff, (3) caregiver-companions’ roles and responsibilities, and (4) the risks to which caregiver-companions can be exposed. Table 1 lists a sample of questions included in the interview guide. Data collection stopped when our target sample size was met, which coincided with the time our recruitment strategies ceased to generate interest from new potential participants.

**Table 1:** Selected interview questions

***Question***	***Sub-probes***
What do you see as your role with regard to travel companions?	- How much interaction do you typically have with patients’ travel companions?
	- What are some of the reasons that you interact with travel companions?
What kinds of responsibilities do you commonly see travel companions taking on?	- How prepared do you think travel companions are to take on these various responsibilities?
	- Are there any things you think could be done to assist with preparing them for these responsibilities?
What health and safety risks, including stressors, do travel companions face?	- Which of these risks has the most significant impact?
	- Who assists travel companions with minimizing these risks?

### Analysis

All interviews were recorded digitally and transcribed verbatim. Thematic analysis was employed, which involved six steps. First, the transcripts were review by all authors. Second, the team met face-to-face to discuss emerging themes as well as outliers. Third, themes that were collectively agreed upon were used to create a preliminary coding scheme by the first and second authors. The coding scheme identified umbrella concepts and their components into which data segments were categorized to inform thematic analysis [35]. Fourth, transcripts were coded by the first author using NVivo qualitative data management software, with input on code refinement being sought from the second author. Fifth, emerging trends and patterns relevant to the themes pursued in the current analysis were identified in the coded data by the first and second authors. Sixth, these trends and patterns were then compared to existing knowledge and the study objectives by the first and second authors and confirmed by the full team to reveal a refined interpretation of meaning in coded data [36]. Importantly, common themes emerged through this process despite differences in participant work environments and work histories, which is a characteristic of thematic analysis [37].

## Results

We conducted 20 interviews with 21 IPCs (one interview had two participants). The participants worked at 16 different facilities across Bolivia, Costa Rica, Barbados, Mexico, the United States, Croatia, India, Israel, Thailand and Turkey. Twelve of these facilities often received medical tourists from North American, six commonly saw Europeans, one saw mostly Australians, another saw mostly Africans, and two did not have a dominant region of origin for medical tourists. Some specialized in procedures such as cosmetic surgery, bariatric surgery, orthopedic surgery, oncology procedures, spinal surgeries, veinoplasty, and cardiac surgery, while others provided a myriad of procedures.

At the time of the interview, the length of time participants had been IPCs ranged from six months to 12 years. Five participants worked for companies that were based outside of the hospitals or clinics that they coordinated care for, 12 worked for independent facilities, and four worked for hospital chains. Six were the only IPC employed by their company or facility while 15 worked in IPC teams. The IPCs we interviewed took on a range of responsibilities towards medical tourists. For example, three commonly helped the patient determine an appropriate surgeon and facility, thirteen coordinated the itinerary (e.g., surgical date, days of hospital stay, local accommodations, etc.) and ground transportation (e.g., transport to and from the airport, local transport for the caregiver-companion), fourteen routinely provided education to the patient and the companion, two provided some limited nursing care to the patient, and all answered questions and addressed requests.

In the remainder of this section we discuss the three themes that emerged from the dataset regarding the challenges that IPCs, international facilities, and medical tourists can face when caregiver-companions accompany a medical tourist, namely that: (1) companions require time, attention and resources, (2) companions can disrupt the provision of quality care, and (3) companions can be exposed to risks. It should be noted that, according to the majority of IPCs, the help and companionship that caregiver-companions commonly provide outweigh these challenges and caregiver-companion accompaniment is therefore strongly recommended. We have included verbatim quotations throughout the section in order to enable the participants to ‘speak’ to these thematic findings. Following each quote we indicate the country in which the IPC participant was based as well as the number of years they had worked in this capacity.

### Caregiver-companions can require time, attention, and resources

IPCs pointed out several ways in which the involvement of a caregiver-companion can serve to burden medical tourists. First, “*the costs are bigger when you travel with somebody of course, double airplane ticket, double accommodation, double meals, everything*” (Croatia, 2.5). In other words, there is a financial cost to bringing a caregiver-companion. Second, a caregiver-companion may unintentionally increase the time needed for recovery by creating additional stress for the patient. This can be a consequence of worry or anxiety because *“…if the [caregiver-companion] worries a lot then the patient is getting worried too and as you know the emotional state of the patient is really important for their recuperation process or their healing process…*” (Costa Rica, 0.6). Third, caregiver-companions can further be burdensome by doing nothing at all. In these cases, the companions accompany the patient to the destination country, but are otherwise absent from the medical tourism experience. One participant commented that some of “*the companions they feel that they are here on holiday so the patient is at the hospital themselves*” (Thailand, 6). Participants suspect that this neglectfulness is not intentional; rather, the companions do not understand the full scope of what is expected of them.

Although the patient is the focus of IPCs’ responsibilities, in nearly all cases coordinators also reported the need to attend to caregiver-companions and in doing so divert some of their time and attention away from the medical tourist. For example, they provide guidance when needed. If the companion’s presence is stressful to the patient, they may suggest a tourist activity: “*…in the event that we find that the patient is uncomfortable… we try to, to get… the companion to go somewhere [like]… an island tour or a nice restaurant*” (Barbados, 2). More often, IPCs will need to educate caregiver-companions regarding what to expect during the medical tourism process and what roles they should play. Education is an integral part of the participants’ self-identified role towards the companions. It is often cited as a strategy to avoid stress from the medical tourist, the companion, and the facility staff: “*the more educated the companion, [the] better [it is] for all of us*” (Turkey, 1). The companion may initiate this education by asking questions. One participant noted that companions are “*always very, very worried, full of… maybe unnecessary questions… most often the companions of the patient are more worried and more, more, more curious than the patient himself* ” (Croatia, 2.5). Some caregiver-companions also voice complaints about the facility, the doctor, or about the burden of their own worries. In the latter case, IPCs comfort them: “*…you’re dealing with an emotionally charged individual to begin with and they have different expectations… and it takes a great deal of time and attention to calm them down*” (Thailand, 12). Like educating the companion, many IPCs saw offering comfort as strategically important to a positive experience and, although doing so took time, there were eventual benefits to be had from this time investment.

Caregiver-companions can create a redundancy of some roles with staff in medical tourism facilities. Many perform the hands-on care normally done by nurses, such as by helping the medical tourist with every day tasks after surgery while still in the facility. “*They assist the patient to get up and go to the bathroom, even though the nurses are available to do that… The same as showering, bathing, the nurse is completely capable of doing that but a lot of times the travel companion wants to do that instead*” (Mexico, 12). They also often help with recovery exercises, namely for mobility, and monitor the patient for symptom changes. The responsibility of offering comfort to the patient is sometimes adopted by the companion instead of facility staff because they are familiar and usually constantly at the patient’s side. Caregiver-companions will also communicate with loved ones back home, a task that many IPCs are prepared to do. One participant said that “*some have their own GATT connection or Skype in their computers. And so [I say] ‘I can make an international call for you’. And they say ‘No, I don’t need that. I brought my own equipment. I brought my Skype, I brought my jack, I brought this. I came prepared’*” (Costa Rica, 5). Although these redundancies are seemingly helpful in that they free up the time of facility staff, IPCs indicated that they can pose challenges to continuity in symptom monitoring as well as record keeping.

### Caregiver-companions can disrupt the provision of quality care

Workers at medical tourism hospitals and clinics, and IPCs specifically, can find it challenging to accommodate companions because they do not always respect facilities’ norms. For example, facility rules occasionally need to be changed in light of disruptions the companions introduce to the organizational routine. According to one participant, their facility needed to change the protocol regarding the number of visitors that patients could have in their room at once. The change occurred after the arrival of a patient’s seven companions who “*overran the facility*” (USA, 8). Multiple participants noted that companions often complain about the food on behalf of the patient. In one case, a companion insisted that s/he prepare the patient’s food in the facility’s kitchen. As the participant recounts: “*we have even allowed the attendants of the companion to enter the kitchen and prepare their own kind of food with their own hands*” (India, 2.25). While it is the norm that caregiver-companions provide support and some hands-on care to the patient, in some instances this was not done. For example, in some cases the companions “*passively support*” (Thailand, 6) patients, meaning that they are physically present but they do not adopt any other caregiving responsibilities. Other companions are perceived as uncaring and absent from the bedside. These two types of disruption, exceptional caregiver-companion actions (e.g., insisting on preparing food for the patient) as well as unexpected inaction (e.g., offering only passive support), both create more work for facility staff and disrupt facility norms.

Facilities are sometimes faced with companions who enable patients to go against the advice given by staff and clinicians. Prior to arrival in the destination country, it is important for the patients to follow instructions in preparation for the surgery or to provide accurate information about their health status. This advice is, however, not always followed. Participants noted that in some cases caregiver-companions were complicit in this problematic behaviour. Then, in cases where physicians cancel the procedure as a result, companions can become upset (Thailand, 12):

I: Are there any exceptional or unusual cases of companions accompanying medical tourists that stick out in your mind?

P: Yes there are several, usually it’s misinformation from the patient side. Patients who are HIV positive, who… [have] other underlying problems and they present for a simple procedure and you find out that they have a heart condition and many other things, there are major problems and you can’t treat them… So you turn them away [after they have arrived].

I: …How do the companions react to this?

P: …Often outrage, because they’ve [caregiver-companions] been part [of it]…they [patients] think they can go to another country and get treatment for various things they’ve probably been turned down for in their own country.

Other caregiver-companions may believe that their competence is equal or superior to that of the nurses and physicians, and as a result do not act on clinical advice. This can present as over-protectiveness: “*sometimes the travel companion can be a little bit too over-protective and they think they know better than the doctor*” (Mexico, 1.5 & 0.75). They may also enable patients to ignore or act against facility rules such as those regarding smoking on the hospital or clinic grounds. Thus, caregiver-companions can enable patients to ignore rules and clinical instructions, and also avoid following them themselves.

Some IPCs reported on cases in which caregiver-companions were required to make decisions on clinical advice on behalf of the medical tourists. In the cases of caregiver-companions with limited financial resources, the financial affordability of medical care may inform decision-making and can override clinical advice and best practice, particularly in light of complications that require quick decisions and significant financial resources. One participant told the story of a caregiver companion he worked with (Turkey, 1.5):

Somebody’s dad is in the ICU [intensive care unit] and…there were complications. You have to extend your dad’s stay in the ICU, you don’t know what’s going to happen, and so cost just increases and increases. But you never really thought of …such a long stay and such an expensive hospital particularly in the ICU which… without insurance is a tremendous cost. And so it was a very difficult situation for them I think both financially, emotionally: and ‘how far do you go, how much is your dad worth?’ that was really the question.

Caregiver-companions with limited financial resources may be faced with unanticipated financial challenges on top of the already debilitating cost of medical tourism.

### Caregiver-companions can be exposed to risks

According to the IPCs we spoke with, caregiver-companions can be exposed to stress, which is a health risk, at a number of points throughout the course of a medical tour. The majority of participants noticed that before the patient has had surgery, companions typically experience stress from worry about the outcome of the procedure. “*I would say a little bit stressful… Once the patient’s out and everything is okay they seem to be okay*” (Thailand, 1.5). Another reason for the stress is a lack of trust in the country, the facility, and/or the IPC experienced by some caregiver-companions. One participant summed it up as “*…the stress factors [are] the fears, the prejudice, judgments toward the country, towards the hospital, [and] treatment procedures*” (Turkey, 1). However, these stresses usually fade once caregiver-companions gain some understanding of the country and the hospital environment, and, according to many participants, a large part of the IPCs’ interactions with the companions pre-surgery involves fostering this understanding. Participants also noted that during this time, IPCs often try to educate the companions on the procedure and care plan, as well as the ways in which the companions could be helpful to the patient. This helps to remove the stress associated with feeling unprepared, which is common amongst caregiver-companions who “*really have no clue what’s happening*” (Turkey, 1.5). As one participant noted “*when the patient and the companion are informed about what’s going on they’re… they’re more calm*” (Costa Rica, 2.5). Therefore, education and information are used to minimize or avoid stress.

After surgery, distinctive types of stresses are commonly experienced among caregiver-companions. Multiple participants reported emotional strain in companions due to worry for the patient, stress over an unknown outcome, and uncertainty of their role in assisting with the patients’ pain management and overall wellbeing. One participant noted that “*sometimes [we] will actually have a companion that would be more on verge of a breakdown than the patient because they take such responsibility in the whole situation, wanting everything to be perfect, wanting the patient not to be in pain*” (Thailand, 6). It may also be difficult for caregiver-companions to handle the sight of swelling and surgical wounds immediately after a surgery, especially one that is cosmetic or bariatric in nature. However, the inability to handle this among caregiver-companions can create more stress for the patients and can slow their healing process. Therefore IPCs who work in facilities that offer cosmetic and bariatric procedures exclusively commonly advise patients to travel unaccompanied (Bolivia, 7):

Companions bring more stress to [patients]… Let’s say, one of the stitches comes out early and you have never seen open stitches before, and… you can see inside the [patient] and they get really scared. So [companions] make the patient more stressed out than if [companions] are not there [with them]…. We actually do not advise them to come with a companion.

Many participants characterized companions as worried, and therefore at risk of experiencing stress, because they are constantly anxious about the patient’s welfare. Although the stresses may overlap, there are distinctions between pre- and post-surgery stresses. Before surgery, as discussed in the last paragraph, companions are concerned about trusting their environment as well as their own competencies, which can cause stress. After surgery, their focus tends to be strongly focused on the patients, which can lead to worry and thus mental stress and anxiety.

Companions can face safety risks, some of which can also threaten their health, while abroad. Participants noted that companions and medical tourists alike may be robbed, financially exploited, get lost in the city, or drink contaminated tap water. IPCs will sometimes educate caregiver-companions to about the potential travel risks to help mitigate them for the companion as well as the patient (Croatia, 2.5):

*During the stay in Croatia they can be robbed… they can get lost in the different countries… the patient coordinator will instruct the companion what to do…how to behave in certain countries because the cultures are different, the things are different in each country… so I think the communication between the coordinator and the companion and then with the patient is crucial*.

Risks associated with everyday life are also present while caregiver-companions are abroad. Participants recounted their experiences with companions who were in a bar fight, had fallen down a flight of stairs, were in a car accident, experienced a heart attack, and become ill with a stomach flu while abroad. One participant said: “*we had a companion who went on a hike and broke her leg*” (Israel, 3). Given the range of risks that caregiver-companions may be exposed to while abroad, both within the facility and beyond, it is impossible for IPCs to anticipate the full scope of risks.

## Discussion

The findings shared above show that, according to IPCs who have spent many hours interacting with and addressing challenges posed by numerous caregiver-companions throughout their careers, caregiver-companions and the practice of informal caregiving can present multiple challenges to international medical tourism facilities, their staff, and even to medical tourists. Despite this, the IPCs we spoke with were overwhelmingly supportive of caregiver-companions accompanying medical tourists abroad, with the exception of some who worked with patients receiving bariatric or cosmetic surgeries, because caregiver-companions were perceived to be more helpful overall than challenging. However, they do present challenges. The IPCs pointed out that caregiver-companions may, for example, have a negative impact on the patient through cost of accompaniment or inadequate care provision. Caregiver-companions may also create unanticipated or extra work for IPCs and other workers in destination facilities, as additional clients and by ignoring established organizational rules, routines, and expectations. Furthermore, caregiver-companions may be susceptible to stresses and health and safety risks, which can further deteriorate their own abilities to offer the patient quality care. Our findings show that IPCs believe companions are more successful caregivers and less of a burden to staff when they are receptive to education from IPCs and willing to acting upon it. Our findings also show that the presence of a caregiver-companion can shift the IPC’s focus from the patient to the companion at times. Building on these findings, in this section we contrast what this study has found about the challenges that caregiver-companions can pose against findings in the existing informal caregiving and medical tourism literatures. Doing so enables us to offer some insight into what is distinct about transnational informal caregiving in the medical tourism context.

Our analysis has revealed that caregiver-companions require additional resources. For example, medical tourists often shoulder the financial burden of an extra flight, accommodation and meals when they travel accompanied. For that reason, cost can be an issue of relevance to caregiver-companions’ involvement in medical tourism, a point that was voiced by most IPCs we spoke with. Although other studies have shown that cost can be an important factor in medical tourists’ decision-making [12,38-43] because it may be less costly to obtain a procedure abroad than at home [42,44,45], discussion of cost savings in medical tourism rarely mention the costs associated with bringing a caregiver-companion. In other words, the cost incentive associated with obtaining medical care abroad may be brought into question for those accompanied by a caregiver-companion; a point that is paralleled in some other medical tourism studies that have emphasized the heavy financial burden that can be placed on medical tourists and their families, and particularly those traveling from the Global South [9,46]. Our analysis further shows that caregiver-companions with less financial resources are equally motivated by cost when put in the position of having to make decisions based on clinical advice while abroad, especially when complications occur. This finding echoes concern in the existing medical tourism literature about the role that cost plays in medical tourists’ making decisions based on clinical advice [47-49].

Our findings show that when caregiver-companions travel abroad, they may be exposed to risks and stresses derived from the unknown and unfamiliar nature of their surroundings. Caregiver-companions may even be apprehensive about the medical tourism facility and mistrust their standards of care. This is consistent with media reports of a popular schema about poor care quality and standards in developing countries ^a^[50-53]. According to IPCs, such apprehension can lead caregiver-companions to disrupt a facility’s organizational routines due to mistrust or misunderstanding, which can have significant implications for facility staff and even the medical tourist. Caregiver-companions may also hold pre-existing stereotypes or misconceptions about cultural norms that inform their apprehensions. This point is consistent with the findings of other studies that have pointed out that medical tourists may have concerns regarding the “foreignness” of the destination they are travelling to [16,54,55]. Meanwhile, our study also shows that caregiver-companions’ own apprehensiveness over “foreignness” and the unknown may lessen their abilities to address these same concerns held by medical tourist. Related to this, the unfamiliar care environment is a key difference between the contexts of caregiving in medical tourism and more conventional informal caregiving at home. Conventional caregivers are not burdened by the apprehension of “foreignness”, nor are they predisposed to doubt the quality of local facilities where they spend a minority of their time as caregivers. The need to travel to a foreign facility introduces additional challenges for caregiver-companions that can bring about new stresses or amplify existing ones.

The findings of our study show that caregiver-companions are often needy, in that they require time, attention, and resources, and that IPCs attempt to address these needs. IPCs are available by telephone while the companions and patients are abroad, and they are often physically present while the patient is in the hospital to assist with meeting these needs. While the support, advice, and information they provide to caregiver-companions may seemingly present as extra work, the IPCs that we spoke with viewed it as an intervention aimed at minimizing stress, burden, and other negative outcomes for caregiver-companions, the medical tourist, and facility staff. This aspect of the IPC’s role, in relation to supporting the caregiver-companion, is consistent with the findings of other studies that have shown that medical tourism facilities tend to have significant staffing resources and provide a high amount of interpersonal time dedicated to patients [11,56]. Attentiveness towards caregiver-companions by IPCs can take the form of providing guidance, education, and comfort in order to be responsive to their needs. The provision of education in particular may assist with overcoming what is often reported to be the lack of reliable information available to those engaging in medical tourism [57,58]. Although information being shared by an IPC as an educational strategy to meet caregiver-companions’ needs and avoid the onset of stress is unlikely to be neutral, a concern raised about the commonness of industry-generated information being used to inform decisions about medical tourism [46,58,59], the reportedly trusting relationship built between the companion and coordinator may enhance its perceived trustworthiness.

Multiple studies have shown that having access to meaningful formal support for informal caregivers, such as that provided by IPCs in the context of medical tourism, can translate into increased health and wellbeing for the patient [60,61] and caregiver alike [24-26,62]. The findings of our research confirm that, in the experience of our participants, support in the form of dedicated collaboration between the companion and the IPC is necessary to improve patient outcomes and avoid a slowed recovery. However, researchers and policy-makers have been quick to point out that there is a difference between having access to support and the uptake of this same support, and that some informal caregivers are simply not accepting of assistance in any form [63,64]. IPCs seem to be cognizant of this and they offer support actively and intentionally as a strategy to elucidate a better experience for the medical tourist, the companion, and hospital staff. This suggests that caregiver-companions in medical tourism may be equally in need of being prompted to accept support in order to minimize their stress and burden as other types of informal caregivers.

Informal caregivers typically experience more stress when they are not able to effectively collaborate with health professionals [60,64-67]. Similarly, our findings show that when caregiver-companions are thought to be not open to collaborating with medical tourism facility staff, and most specifically the IPC, it is more difficult for them to carry out their caregiving responsibilities and they may find the experience more stressful. Participants, for example, reported that companions can be incapable of or ineffective at helping patients if they are too worried about surgical outcomes or their own care abilities. Worry is also a sign of caregiver burden [26,68,69] and, not surprisingly, some IPCs identified it as a potential source of stress and negative health outcomes for caregiver-companions. Our findings have also shown that worry is very common throughout the companion’s time abroad and can sometimes negatively impact the patient who, according to other medical tourism studies, may already be experiencing stress [55,70,71].

### Directions for future research

While our analysis sheds new and dedicated light on the challenges introduced by caregiver-companions in medical tourism, and also on the roles and responsibilities more generally associated with this stakeholder group, we believe that much research remains to be done about the intersection between medical tourism and informal caregiving. Our findings have shown some differentiation between caregiver-companions as a result of socio-economic status and the procedure the patient is obtaining abroad, but it is unclear if there are meaningful differences between them based on their relationship with the medical tourist, their country of origin, or the destination country, and what these differences may be. Meanwhile, some of these differences have been found to be extremely important to caregiver health status and patient outcomes in other forms of informal caregiving [72,73]. Our analysis also suggests that the presence of caregiver-companions and the actions they undertake can be beneficial to the health of the patient, and that support from IPCs is beneficial to caregiver-companions and thus ultimately the patient. However, we require more knowledge regarding the other forms of support caregiver-companions draw from or that they require. For example, would caregiver-companions benefit from gaining access to some or all of the interventions developed for informal caregivers in various health systems and governments, such as system navigation tools, support groups, and therapeutic interventions [31,74,75]? This is a pressing question that researchers can assist with answering.

This analysis helps to demonstrate the value in speaking with stakeholders in medical tourism who are knowledgeable about other stakeholder groups, and particularly groups that are difficult to locate (as is the case with caregiver-companions). In particular, it highlights the potential for having IPCs serve as participants in medical tourism research. Our findings have shown IPCs’ perspectives on the challenges introduced by caregiver-companions, but a counter-point to this, examining the benefits they introduce for IPCs and other destination facility staff would be a useful direction for future research. Our findings have further shown that the extra expense of travelling abroad with a friend or a family member may discourage some medical tourists from taking a companion. However, research has hinted at the regularity with which friends and family members accompany medical tourists abroad [9-11]. A detailed account of the reasons for which they are perceived as useful enough to justify the extra expense has yet to be undertaken. IPCs would be ideal participants for such a study for the same reasons that we found them helpful for our own: they have the most interaction with the highest number of companions out of any stakeholder group that we are aware of and they can therefore speak to the breadth and depth of a particular issue relevant to those they interact with in a professional capacity.

### Limitations

First, recruitment information indicated that interviews could be conducted in French and English. Upon request of an interested potential participant, we were able to coordinate a Spanish-language interview as well. IPCs who were not fluent in these languages were not included in this study. Second, the utilization of semi-structured phone and Skype interviews may have resulted in some missed data due to the inaccessibility of visual cues and the formality of the conversation. Studies have shown, however, that phone interviews are a reliable and cost effective method for data collection [76], and for this reason we are confident in the soundness of the dataset. Third, in this analysis we have considered many aspects of the caregiver-companion’s experience in medical tourism, yet we did not consult with a single caregiver-companion in doing so. While we believe that our use of IPCs to gather information about this stakeholder group is well justified, it is nonetheless a limitation that we present information about caregiver-companions here solely from the perspective of IPCs. A clear direction for future research, including our own, is to speak with caregiver-companions themselves about the experiences embedded in the current analysis along with other aspects of their informal caregiving roles and responsibilities.

## Conclusions

In this article, we presented the findings of a thematic analysis derived from 20 interviews with 21 IPCs working in 16 medical tourism facilities across nine countries. We examined concerns that these IPCs identified regarding the challenges that caregiver-companions and the practice of informal caregiving may pose to facility staff and medical tourists. These concerns include the potential for caregiver-companions to be exposed to health and safety risks that negatively impact not only themselves but also the medical tourist. It was also shown that caregiver-companions can be burdensome to medical tourism facility staff, including the IPCs, through requiring time and attention in order to minimize or lessen anxiety, worry, and ultimately stress. IPCs tend to actively take steps to mitigate the potential for challenges to arise in relation to informal caregiving in medical tourism, with a particular focus on caregiver-companion education. Our findings suggest that if companions are open to collaboration with IPCs, and particularly in the form of information sharing, then their experience abroad can be safer and less stressful for themselves and, by extension, for the accompanied patients and facility staff.

This article has positioned caregiver-companions as a central stakeholder group in the global health services practice of medical tourism and has also shown the value of asking one stakeholder group, namely IPCs, about their experiences with another, namely caregiver-companions, to gain broader knowledge. Most broadly, the analysis shed light on the very practice of informal caregiving in medical tourism, which is a particular form of transnational caregiving – a form of caregiving that has received little dedicated research attention. Caregiver-companions represent the intersection between medical tourism and informal caregiving and, to our knowledge, this is the first account that focuses on this intersection. We encourage more research to be done so that informal caregiving in medical tourism can be further examined and also so that appropriate responses to and interventions for this transnational caregiving practice can be implemented.

## Endnote

^a^Medical tourists from developing countries might perceive the opposite: the local standard of care might be seen as inferior whereas the foreign one is held on a pedestal [9].

## Competing interests

The authors declare that they have no competing interests.

## Authors’ contributions

VC conducted the interviews, led the coding, and played a leadership role in writing this article. VAC is lead investigator on the study that funded this research and developed the protocol. She contributed to all aspects of the study and was heavily involved in preparing this manuscript. JS and LT contributed input to the data collection instrument, participated actively in the face-to-face analysis meeting, and provided feedback on drafts of this paper. JS also aided in recruitment. All authors reviewed and approved the submitted manuscript.

## References

[B1] Connell J Contemporary medical tourism: Conceptualisation, culture and commodification Tourism Manage 2013 34 1 13

[B2] Lunt N Smith R Exworthy M Green ST Horsfall D Mannion R Medical tourism: Treatments, markets and health system implications: A scoping review OECD 2011 http://www.oecd.org/els/health-systems/48723982.pdf

[B3] Ehrbeck T Guevara C Mango PD Cordina R Singhal S Health care and the consumer McKinsey Quart 2008 4 80 91

[B4] Mattoo A Rathindran R How health insurance inhibits trade in health care Health Aff (Millwood) 2006 25 358 368 10.1377/hlthaff.25.2.358 16522578

[B5] Carrera P Bridges J Globalization and healthcare: understanding health and medical tourism Expert Rev Pharmacoecon Outcomes Res 2006 6 447 454 10.1586/14737167.6.4.447 20528514

[B6] Eggertson L Wait-list weary Canadians seek treatment abroad Can Med Assoc J 2006 174 1247 10.1503/cmaj.060219 16636318 PMC1435972

[B7] Milstein A Smith M America’s new refugees – seeking affordable surgery offshore New Engl J Med 2006 355 1637 40 10.1056/NEJMp068190 17050886

[B8] Hopkins L Labonte R Runnels V Packer C Medical tourism today: What is the state of existing knowledge J Public Health Pol 2010 31 185 198 10.1057/jphp.2010.10 20535101

[B9] Kangas B Hope from abroad in the international medical travel of Yemeni patients Anthropol Med 2007 14 293 305 10.1080/13648470701612646 27268744

[B10] Yeoh E Othman K Ahmad H Tourism Management Understanding medical tourists: Word-of-mouth and viral marketing as potent marketing tools Tourism Manage 2013 34 196 201

[B11] NaRanong A NaRanong V The effects of medical tourism: Thailand's experience Bull World Health Organ 2011 89 336 344 10.2471/BLT.09.072249 21556301 PMC3089382

[B12] Yu JY Ko TG A cross-cultural study of perceptions of medical tourism among Chinese, Japanese and Korean tourists in Korea Tourism Manage 2012 33 80 88 10.1016/j.tourman.2011.02.002

[B13] Crooks VA Snyder J Johnston R Kingsbury P Perspectives on Canadians’ involvement in medical tourism: Final research report [http://www.sfu.ca/medicaltourism/Perspectives_on_Canadians_Involvement_in_Medical_Tourism.pdf]23870781

[B14] Kingsbury P Crooks VA Snyder J Johnston R Adams K Narratives of emotion and anxiety in medical tourism: on *State of the Heart* and *Larry's Kidney* Soc Cult Geogr 2012 13 361 378 10.1080/14649365.2012.683807

[B15] Rose DA Larry's Kidney: Being the True Story of How I Found Myself in China with My Black Sheep Cousin and His Mail-Order Bride, Skirting the Law to Get Him a Transplant–and Save His Life 2009 New York: William Morrow

[B16] Grace M State of the Heart: A Medical Tourist's True Story of Lifesaving Surgery in India 2007 Oakland, CA: New Harbinger Publications

[B17] Medical Tourism Association Patient Survey [http://www.medicaltourismassociation.com/userfiles/files/Patient%20Survey-FINAL.PDF]

[B18] Travers R Thelen S Helmy E Nabil N Elmasri AW Medical tourism development strategy final report [http://www.imcegypt.org/studies/FullReport/Medical%20Tourism%20Development%20Strategy_EN.pdf]

[B19] Molloy GJ Johnston DW Witham MD Family caregiving and congestive heart failure: Review and analysis Eur J Heart Fail 2005 7 592 603 10.1016/j.ejheart.2004.07.008 15921800

[B20] Bevan JL Pecchioni LL Understanding the impact of family caregiver cancer literacy on patient health outcomes Patient Educ Couns 2008 71 356 364 10.1016/j.pec.2008.02.022 18372142

[B21] Wright AA Keating NL Balboni TA Matulonis UA Block SD Prigerson HG Place of death: Correlations with quality of life of patients with cancer and predictors of bereaved caregivers' mental health J Clin Oncol 2010 28 4457 4464 10.1200/JCO.2009.26.3863 20837950 PMC2988637

[B22] Cook JA Cohler BJ Pickett SA Beeler JA Life-Course and Severe Mental Illness: Implications for Caregiving Within the Family of Later Life Fam Relat 1997 46 427 436 10.2307/585102

[B23] Mittelman MS Haley WE Clay OJ Roth DL Improving caregiver well-being delays nursing home placement of patients with Alzheimer disease Neurology 2006 67 1592 1599 10.1212/01.wnl.0000242727.81172.91 17101889

[B24] Carretero S Garces J Rodenas F Sanjose V The informal caregiver’s burden of dependent people: Theory and empirical review Arch Gerontol Geriat 2009 49 74 79 10.1016/j.archger.2008.05.004 18597866

[B25] Hawranik PG Strain LA Giving voice to informal caregivers of older adults Can J Nurs Res 2007 39 157 172 17450711

[B26] Rocha Pereira H Rebelo Botelho MA Sudden informal caregivers: the lived experience of informal caregivers after an unexpected event J Clin Nurs 2011 20 2448 2457 10.1111/j.1365-2702.2010.03644.x 21676044

[B27] Lee H Singh J Appraisals, Burnout and Outcomes in Informal Caregiving Asian Nurs Res 2010 4 32 44 10.1016/S1976-1317(10)60004-7 25030791

[B28] Borg C Hallberg IR Life satisfaction among informal caregivers in comparison with noncaregivers Scan J Caring Sci 2006 20 427 438 10.1111/j.1471-6712.2006.00424.x 17116152

[B29] Pinquart M Sorensen S Differences between caregivers and noncaregivers in psychological health and physical health: A meta-analysis Psychol Aging 2003 18 250 267 12825775 10.1037/0882-7974.18.2.250

[B30] Roth DL Perkins M Wadley VG Temple EM Haley WE Family caregiving and emotional strain: Associations with quality of life in a large national sample of middle-aged and older adults Qual life res 2009 18 679 688 10.1007/s11136-009-9482-2 19421895 PMC2855243

[B31] Commission on the Future Health Care in Canada Building on values: The future of health in Canada [http://publications.gc.ca/collections/Collection/CP32-85-2002E.pdf]

[B32] Friedman B Wamsley BR Liebel DV Saad ZB Eggert GM Patient satisfaction, empowerment, and health and disability status effects of a disease management: Health promotion nurse intervention among medicare beneficiaries with disabilities Gerontologist 2009 49 778 792 10.1093/geront/gnp090 19587109

[B33] Martínez Álvarez M Chanda R Smith RD The potential for bi-lateral agreements in medical tourism: A qualitative study of stakeholder perspectives from the UK and India Global Health 2011 7 11 10.1186/1744-8603-7-11 21539738 PMC3110115

[B34] Birch DW Vu L Karmali S Johnson Stoklossa C Sharma AM Medical tourism in bariatric surgery Am J Surg 2010 199 604 608 10.1016/j.amjsurg.2010.01.002 20346442

[B35] Schwandt TA Thematic analysis The SAGE Dictionary of Qualitative Inquiry 2007 3 Thousands Oaks, CA: SAGE Publications Inc 292

[B36] Fereday J Muir-Cochrane E Demonstrating rigor using thematic analysis: A hybrid approach of inductive and deductive coding and theme development Int J Qual Meth 2006 5 80 92

[B37] Braun V Clarke V Using thematic analysis in psychology Qual Res Psychol 2006 3 77 101 10.1191/1478088706qp063oa

[B38] Leng CH Medical tourism in Malaysia: international movement of healthcare consumers and the commodification of healthcare Asia Res Inst 2007 83 1 32

[B39] Horowitz MD Rosensweig JA Medical tourism: health care in the global economy Phys Exec 2007 33 24 30 18092615

[B40] Garud AD Speaking for ourselves: Medical tourism and its impact on our healthcare Natl Med J India 2005 18 318 319 16483034

[B41] Lindsay G Medical leave. Fast Comp 2008 125 108 119

[B42] Glinos IA Baeten R Boffin N Rossenmöller M, McKee M, Baeten R Cross-border contracted care in Belgium hospitals Patient mobility in the European Union: learning from experience 2006 Copenhagen, Denmark: European Observatory on Health Systems and Policies 97 118

[B43] Cohen IG Protecting patients with passports: Medical tourism and the patient protective- argument Iowa Law Rev 2010 95 1467 1567

[B44] The Gallup Organization Cross-border health services in the EU[http://ec.europa.eu/public_opinion/flash/fl_210_en.pdf]

[B45] Burkett L Medical tourism: Concerns, benefits, and the American legal perspective J Legal Med 2007 28 223 245 10.1080/01947640701357763 17558794

[B46] Johnston R Crooks VA Snyder J “I didn’t even know what I was looking for”: A qualitative study of the decision-making processes of Canadian medical tourists Global Health 2012 8 23 10.1186/1744-8603-8-23 22769723 PMC3475067

[B47] Cheung IA Wilson A Arthroplasty tourism Med J Aust 2007 187 666 667 18072912 10.5694/j.1326-5377.2007.tb01467.x

[B48] Turner L ‘First world health care at third world prices’: Globalization, bioethics and medical tourism Biosocieties 2007 2 303 325 10.1017/S1745855207005765

[B49] Turner L (2007b) Medical tourism: Family medicine and international health-related travel Can Fam Physician 2007 53 1639 1641 17934018 PMC2231416

[B50] Casacchia C Medical tourism’ gains steam as health care costs rise in US[http://www.bizjournals.com/phoenix/stories/2010/05/03/story7.html]23890934

[B51] Meyer B Medical tourism: Lured by stem cells' promise, Americans head abroad for medical treatment[http://www.cleveland.com/world/index.ssf/2009/02/medical_tourism_lured_by_promi.html]

[B52] Russell R Surgery in the sun lures patients to Thailand [http://uk.reuters.com/article/2007/03/07/uk-thailand-medical-idUKSP13935820070307]

[B53] Martin R Clark Howard Discusses His Cancer Diagnosis With Dr. Randy Martin [http://www.wsbtv.com/news/news/clark-howard-discusses-his-cancer-diagnosis-with-d/nFBhr/]

[B54] Mudur G Hospitals in India try and woo patients BMJ 2004 328 1338 1338 15178611 10.1136/bmj.328.7452.1338 PMC420282

[B55] Howze KS Medical tourism: symptom or cure? Georgia L Rev 2007 41 1013 1052

[B56] Connell J Medical tourism: Sea, sun, sand and … surgery Tourism Manage 2006 27 1093 1100 10.1016/j.tourman.2005.11.005

[B57] Lunt N Carrera P Medical tourism: Assessing the evidence on treatment abroad Maturitas 2010 66 27 32 10.1016/j.maturitas.2010.01.017 20185254

[B58] Lunt N Hardey M Mannion R Nip, tuck and click: Medical tourism and the emergence of web-based health information Open Med Inform J 2010 4 1 11 10.2174/1874431101004010001 20517465 PMC2874214

[B59] Eysenbach G An ontology of quality initiatives and a model for decentralized, collaborative quality management on the (semantic) world wide web J Med Internet Res 2001 3 34 10.2196/jmir.3.4.e34 PMC1761914 11772549

[B60] Northouse L Williams AI Given B McCorkle R Psychosocial care for family caregivers of patients with cancer J Clin Oncol 2012 30 1227 1234 10.1200/JCO.2011.39.5798 22412124

[B61] Surbone A Baider L Are oncologists accountable only to patients or also to their families? An international perspective 2012 Alexandria, VA: American Society of Clinical Oncology 10.14694/EdBook_AM.2012.32.307 24451822

[B62] Mangan PA Taylor KL Yabroff KR Fleming DA Ingham JM Caregiving near the end of life: Unmet needs and potential solutions Palliat Support Care 2003 1 247 259 16594425 10.1017/s1478951503030414

[B63] Bass D Noelker LS Rechlin LR The moderating influence of service use on negative caregiving consequences Gerontol B Psychol Sci Soc Sci 1996 51B S121 S131 10.1093/geronb/51B.3.S121 8620359

[B64] Weinberg DB Lusenhop RW Hoffer Gittell J Kautz CM Coordination between formal providers and informal caregivers Health Care Manage Rev 2007 32 140 149 10.1097/01.HMR.0000267790.24933.4c 17438397

[B65] Bucher JA Loscalzo M Zabora J Houts PS Hooker C BrintzenhofeSzoc K Problem-solving cancer care education for patients and caregivers Cancer Pract 2001 9 66 70 10.1046/j.1523-5394.2001.009002066.x 11879281

[B66] Morse JM Pooler C Patient-family-nurse interactions in the trauma-resuscitation room Am J Crit Care 2002 11 240 249 12022487

[B67] Egerod I Overgaard D Taking a back seat: support and self-preservation in close relatives of patients with left ventricular assist device Eur J Cardiovasc Nurs 2012 11 380 387 10.1177/1474515111435609 22514140

[B68] Jacobs B Levine C From sadness to pride: seven common emotional experiences of caregiving Always on Call: When Illness Turns Families into Caregivers 2004 Nashville: Vanderbilt University Press 111 125

[B69] Wolpe G Levine C (2004) A crisis of caregiving, a crisis of faith Always on Call: When Illness Turns Families into Caregivers 2004 Nashville: Vanderbilt University Press 34 44

[B70] Law J Sun, sand and stitches Profit 2008 27 69 70

[B71] Lautier M Export of health services from developing countries: The case of Tunisia Soc Sci Med 2008 67 101 110 10.1016/j.socscimed.2008.01.057 18468756

[B72] Janevic MR Connell CM Racial, Ethnic, and Cultural Differences in the Dementia Caregiving Experience Gerontologist 2001 41 334 347 10.1093/geront/41.3.334 11405431

[B73] Dentinger E Clarkberg M Informal caregiving and retirement timing among men and women: Gender and caregiving relationships in late midlife J Fam Issues 2002 23 857 879 10.1177/019251302236598

[B74] Rizzo VM Gomes A Chalfy A Using an aging services agency/university partnership to study informal caregiver respite programs Ageing Int 2013 38 71 81 10.1007/s12126-012-9172-1

[B75] World Federation for Mental Health Caring for the caregiver: Why your mental health matters when you are caring for others [http://www.wfmh.org/PDF/Caring%20for%20the%20Caregiver%2011_04_09%20FINAL%20(3).pdf]

[B76] Laws S Harper C Marcus R Telephone interviews Research for Development 2003 Thousand Oaks, CA: SAGE 298 299

